# Diagnosis and Management of Group a Streptococcal Pharyngitis in the United States, 2011–2015

**DOI:** 10.1186/s12879-019-3835-4

**Published:** 2019-02-26

**Authors:** Robert Luo, Joanna Sickler, Farnaz Vahidnia, Yuan-Chi Lee, Bianca Frogner, Matthew Thompson

**Affiliations:** 1 Roche Molecular Systems, Inc, 4300 Hacienda Drive, Pleasanton, CA 94588 USA; 2 Roche Diagnostics Information Solutions, 4300 Hacienda Drive, Pleasanton, CA 94588 USA; 3 Primary Care Innovations Lab, Department of Family Medicine, UW Northgate Clinic, University of Washington, Box 354696, 314 NE Thornton Place, Seattle, WA 98195 USA

**Keywords:** Streptococcal pharyngitis, NAAT, Diagnostics, Antibiotic use

## Abstract

**Background:**

Clinical guidelines for the diagnosis of group A streptococcal (GAS) pharyngitis recommend the use of a rapid antigen detection test (RADT) and/or bacterial culture. This study evaluated the overall diagnosis and treatment of acute pharyngitis in the United States, including predictors of test type and antibiotic prescription.

**Methods:**

A retrospective analysis of pharyngitis events from 2011 through 2015 was conducted using the MarketScan commercial/Medicare databases. A pharyngitis event was defined as occurring within 2 weeks from the index visit. Patient and provider characteristics were examined across 5 testing categories: RADT, RADT plus culture, other tests, nucleic acid amplification testing (NAAT), and no test. Multivariate models were used to identify significant predictors of NAAT use and antibiotic prescription.

**Results:**

A total of 18.8 million acute pharyngitis events were identified in 11.6 million patients. Roughly two-thirds of events (68.2%) occurred once, and roughly a third of patients (29.1%) required additional follow-up, but hospitalization was rare (0.3%). Across all events, 43% were diagnosed by RADT, while 20% were diagnosed by RADT plus culture. The proportion of events diagnosed by NAAT increased 3.5-fold from 2011 to 2015 (0.06% vs 0.27%). Antibiotic use was frequent (49.3%), less often in combination with RADT plus culture (31.2%) or NAAT alone (34.5%) but significantly more often with RADT alone (53.4%) or no test (57.1%). Pediatricians were significantly less likely than other providers to prescribe antibiotics in their patients, regardless of patient age (*p* < 0.0001).

**Conclusions:**

Antibiotic use for sore throat remains common, with many clinicians not following current guidelines for diagnosis of GAS pharyngitis. Diagnosis of GAS pharyngitis using RADT plus culture or NAAT alone was associated with lower use of antibiotics. Diagnostic testing can help lower the incidence of inappropriate antibiotic use, and inclusion of NAAT in the clinical guidelines for GAS pharyngitis warrants consideration.

**Electronic supplementary material:**

The online version of this article (10.1186/s12879-019-3835-4) contains supplementary material, which is available to authorized users.

## Background

Acute pharyngitis is a common medical condition that results in an estimated 15 million healthcare visits per year in the United States [1, 2]. Infection with *Streptococcus pyogenes* (group A beta-hemolytic streptococci) is the most common bacterial cause of acute pharyngitis and is responsible for an estimated 5 to 15% of sore throat cases among adults [3] and 20 to 30% of cases among children [2, 4]. Most cases of pharyngitis will resolve on their own without treatment, however, antibiotics are prescribed in approximately 60% of cases to prevent rare complications (e.g., acute rheumatic fever, rheumatic heart disease, post–streptococcal glomerulonephritis), shorten the duration of illness, prevent the spread of infection to close contacts, and address patient demands [5–7]. Current treatment guidelines discourage the empirical use of antibiotics for sore throats due to concerns about unnecessary antibiotic exposure and development of resistance.

Accurate diagnosis of group A streptococcal (GAS) pharyngitis by clinical symptoms alone is limited due to the overlap of clinical signs and symptoms between bacterial and viral pharyngitis [1]. Current guidelines for diagnosis of GAS pharyngitis in the United States recommend the use of a rapid antigen detection test (RADT) and/or bacterial culture of a throat swab [1]. RADT assays have the benefits of ease of use, rapid turnaround time (< 10 min), and high specificity (95%) but have relatively low sensitivity (70–90%) [8]. As such, negative RADT results require a confirmatory bacterial culture in pediatric patients, patients at high risk of complications from GAS pharyngitis, and any setting in which clinicians wish to maximize diagnostic sensitivity [1, 8]. Bacterial culture is both highly sensitive and specific (90–95%) when performed correctly, but is labor intensive and costly and requires an experienced clinical laboratory to grow and accurately test the bacteria, resulting in reporting delays of 1 to 5 days [9]. Given the low sensitivity rates of RADT and the delays in result reporting (or unavailability) of culture testing, clinicians are often left with the difficult decision of whether or not to prescribe antibiotics when using rapid antigen assays while waiting for confirmatory results, or simply treating (or not treating) the patient without the use of a diagnostic test and accepting any negative clinical consequences or follow-up care.

A number of nucleic acid amplification testing (NAAT) assays for GAS pharyngitis diagnosis have received US Food and Drug Administration (FDA) clearance over the last 3 years. NAAT has shown equivalent sensitivity and specificity to those of bacterial culture [10–15] and improved sensitivity compared with RADT when diagnosing GAS pharyngitis [11]. The potential benefit of using a single assay alone for GAS pharyngitis diagnosis has resulted in some integrated health networks switching to NAAT and implementing rapid transport and reporting mechanisms to reduce turnaround times from days to hours [16]. This facilitates rapid result reporting and timely initiation of appropriate antibiotic therapy, if warranted. Current GAS pharyngitis guidelines do not yet provide guidance on the use of NAAT; however, a recent report from the American Academy of Microbiology suggested that “practice guidelines could inform providers that the nucleic acid tests perform on par with gold standard laboratory testing and encourage their use” [17].

Currently, limited data exist on the factors that impact antibiotic prescribing in GAS pharyngitis. The current study evaluated the diagnosis and treatment of GAS pharyngitis in the United States from 2011 through 2015 in over 11 million patients and investigated the relationship between antibiotic prescribing and provider type, place of service, and GAS pharyngitis diagnostic testing methods. Findings from this study will provide further evidence to inform clinicians and policy makers about which diagnostic tests are routinely used in clinical practice and their impact on antibiotic prescribing for sore throats.

## Methods

Data were compiled from the MarketScan Commercial Claims and Encounters (CCAE) and Medicare Supplemental (MDCR) databases (Truven Health Analytics). The CCAE database contains medical and drug information from employers and health plans, including data on employees and their dependents, whereas the MDCR database includes data on retirees with Medicare supplemental insurance paid by employers [18]. More than 300 employers and 40 private health plans contribute claims information to the MarketScan databases, which is a large-enough data set to provide a nationally representative sample of the American population with employer-sponsored health coverage. The databases have contained data on more than 220 million unique covered individuals from the entire United States since 1995. This analysis used de-identified claims data from inpatient and outpatient visits as well as pharmacies.

**Fig. 1:**
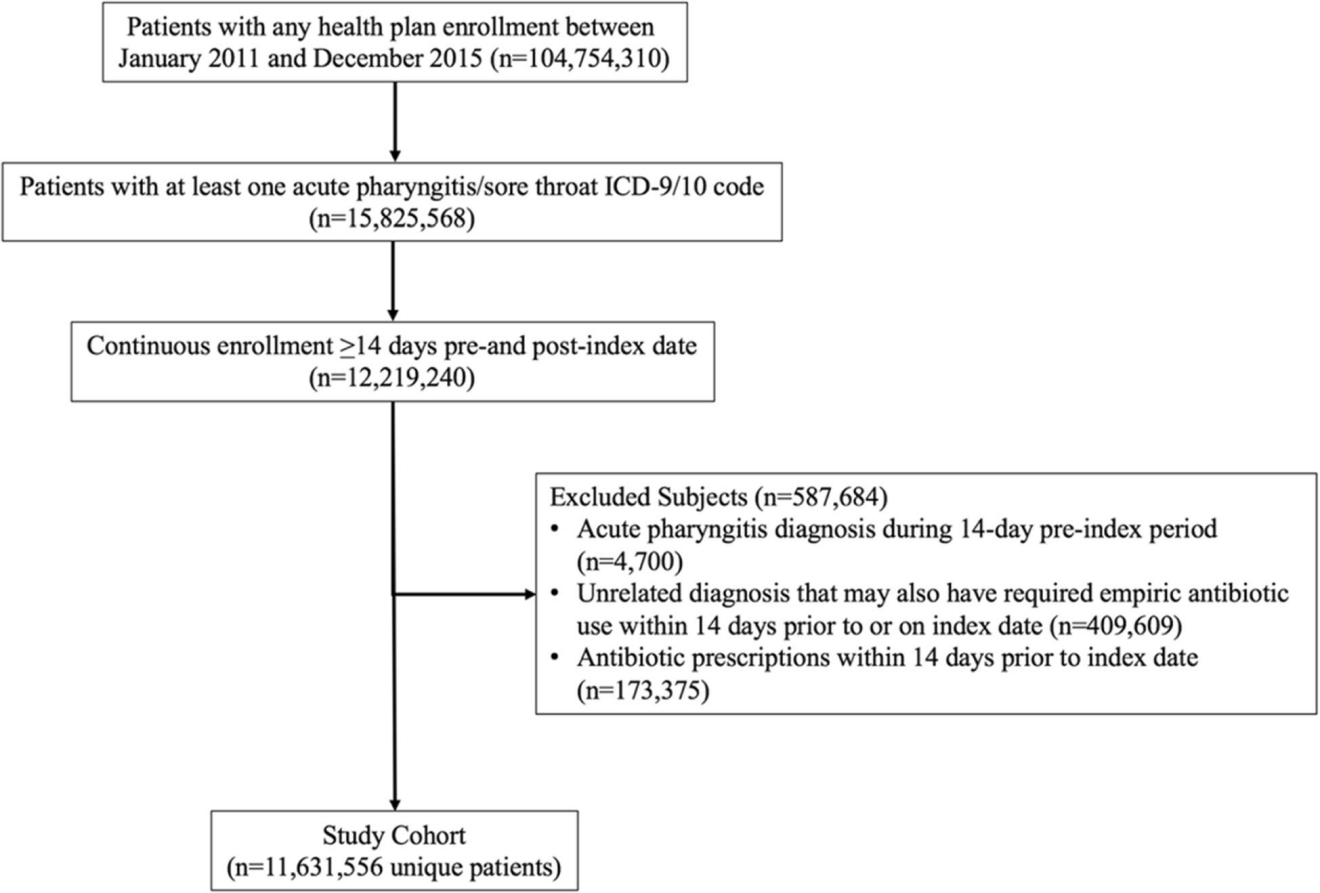
Flow Diagram of Patient Selection Into the Study Cohort

From the databases, all patients with a pharyngitis event between January 1, 2011, and December 31, 2015, were identified. Streptococcal pharyngitis was defined by the presence of at least 1 claim during the study period with any *International Classification of Diseases, Ninth or Tenth Revision, Clinical Modification* diagnostic code for streptococcal sore throat (034.0), acute pharyngitis (462) or acute tonsillitis (463), mapped out to equivalent ICD-10 codes. The index date was defined as the first day of each episode of a pharyngitis claim (index visit) during the study period. A pharyngitis claim (event) was followed for a 2-week period after the index visit (through January 15, 2016) or disenrollment from the insurance plan, whichever came first. If a patient had a second claim for a pharyngitis visit within 2 weeks after the index visit, it was considered a follow-up to the index event. If a patient had a second claim for a pharyngitis visit more than 2 weeks after the index visit, it was treated as a new event in the analysis. Patients were excluded from the analysis if any condition requiring empirical antibiotic use, including acute bronchitis, cystitis, cellulitis, urethritis, pyelonephritis, diverticulitis, and pneumonia, was documented 2 weeks prior to the index visit (Fig. 1) to remove antibiotic prescriptions not related to the index pharyngitis event.

Descriptive analyses were performed to examine patient and pharyngitis event characteristics, both for the entire cohort and stratified adult (age ≥ 18 years) and pediatric (age < 18 years) categories. Patient characteristics examined were sex, geographic region of the United States, health plan type, and number of pharyngitis events. Event characteristics included place of service (categorized in the following order: emergency department > urgent care > physician office > laboratory/other, where the first was used if there were multiple places of service associated with an event), provider type (i.e., pediatrician, family physician, internist, and other/unknown), number of inpatient hospital admissions associated with a pharyngitis event, and medical complications from GAS pharyngitis (i.e., scarlet fever, post–streptococcal glomerulonephritis, rheumatic fever, and streptococcal pneumonia).

GAS pharyngitis diagnostic tests were identified using *Current Procedural Terminology* (*CPT*) codes for NAAT (87651), throat culture (87,081, 87,070, 87,071), RADT (87880), and streptococcal immunoassay (87430). Pharyngitis events were grouped by any GAS pharyngitis diagnostic test claims within 2 weeks after the index visit into the following 5 testing categories: NAAT only, RADT only, RADT plus culture, no test, and other test combination. Other test combination included any combination of the aforementioned test categories with or without streptococcal immunoassay (*CPT* code: 87430). Antibiotic prescription for a pharyngitis event was defined as the presence of a pharmacy claim on or within 2 weeks after the index visit that had a National Drug Code for any of the antibiotics recommended in GAS pharyngitis treatment guidelines [1].

Multivariable models were used to identify pharyngitis event characteristics associated with antibiotic prescription and NAAT use. For each outcome, separate models were constructed for pediatric and adult patients. Adjusted hazard ratios and 95% confidence intervals were calculated using an Anderson-Gill analysis [19] (SAS version 9.4; SAS Institute Inc) in a time-dependent model with the following covariates: sex, region, health plan type, calendar year, place of service and provider specialty. Antibiotic prescription models also included diagnostic test type as a covariate. In all models, 0.5 day was added to the follow-up for all events to avoid exclusion of events when a censoring event occurred on the same day as the index visit.

## Results

A total of 104,754,310 patients were identified in the MarketScan databases between January 1, 2011, and December 31, 2015, of whom 11,631,556 met the criteria for inclusion in the study cohort (Fig. 1). Overall, the study cohort had a mean (SD) age of 24.5 (18.5) years. The majority of patients were female (58.1%), and 54.1% of all patients were ≥ 18 years of age (Table 1). Most patients had only 1 pharyngitis event during the study period (68.2%). When stratified by age group, a higher proportion of adults than children were female (63.7% vs. 51.6%) (Table 1).

**Table 1:** Patient characteristics

Characteristic	All Ages	Age < 18 years	Age ≥ 18 years
No. of patients	11,631,556	5,333,333	6,293,223
Age, mean (SD), years	24.5 (18.5)	8.4 (4.9)	38.0 (14.4)
Sex, %			
Male	41.9	48.5	36.3
Female	58.1	51.6	63.7
Region of the United States, %			
Northeast	17.7	18.1	17.4
North central	22.5	23.3	21.8
South	42.9	42.7	43.0
West	14.7	13.5	15.8
Unknown	2.2	2.4	2.0
Health plan type, %^a^			
Managed care (EPO/HMO)	12.5	12.5	12.5
Preferred provider (POS/PPO)	71.0	70.5	71.5
High deductible (CDHP/HDHP)	11.6	12.0	11.3
Unknown	4.9	5.1	4.7
Distinct acute pharyngitis events, 2011–2015, %			
1	68.2	56.4	78.3
2	18.2	21.7	15.3
3	6.8	10.0	4.2
≥4	6.7	11.9	2.3

^a^Managed care is with an established physician network to receive care: HMO, health maintenance organization, EPO, exclusive provider organization; Preferred provider is with an established physician network and the option to see provider out of the network generally with increased fees: POS, point of service; POS with cap, point of service with capitation; PPO, preferred provider organization; High deductible requires members to pay for services out of pocket until a limit is reached: HDHP, high-deductible health plan. Some include reimbursement from an account before the insurance starts: CDHP, consumer-driven health plan

Within the entire study cohort, there were 18,778,397 distinct pharyngitis events (Table 2). Adults were more likely than children to have only 1 pharyngitis event (78.3% vs. 56.4%). Roughly one-third of events led to 1 or more follow-up visits (29.1%), but hospitalization was rare (0.3%). Overall, antibiotics were used for 49.3% of events and were prescribed at higher rates among adults (54.4%) than among children (45.0%). When information was available on provider specialty type, the most common provider specialties associated with the index visit were pediatrician (28.3%), family physician (26.2%), and internist (7.1%) across all events (Table 2).

**Table 2:** Pharyngitis event characteristics

Characteristic	All Ages	Age < 18 years	Age ≥ 18 years
No. of events	18,778,397	10,229,548	8,548,849
Provider specialty, %			
Pediatrician^a^	28.3	49.9	2.5
Family physician	26.2	15.4	39.0
Internist	7.1	2.3	12.8
Other/unknown	38.4	32.4	45.7
Antibiotics prescribed, %	49.3	45.0	54.4
Follow-up visits, %^b^			
0	70.9	73.7	67.6
1	20.4	19.5	21.3
≥2	8.7	6.8	11.0
Hospital admissions, %			
0	99.7	99.8	99.6
1	0.3	0.2	0.4
≥2	0.01	0.01	0.01
Other complications, %^c^	0.18	0.31	0.03

^a^Primary care (general practice) physician who specializes in children (< 18 years)

^b^Fourteen days post-index visit

^c^Complications related to streptococcal pharyngitis: scarlet fever, post–streptococcal glomerulonephritis, rheumatic fever, and streptococcal pneumonia

**Fig. 2:**
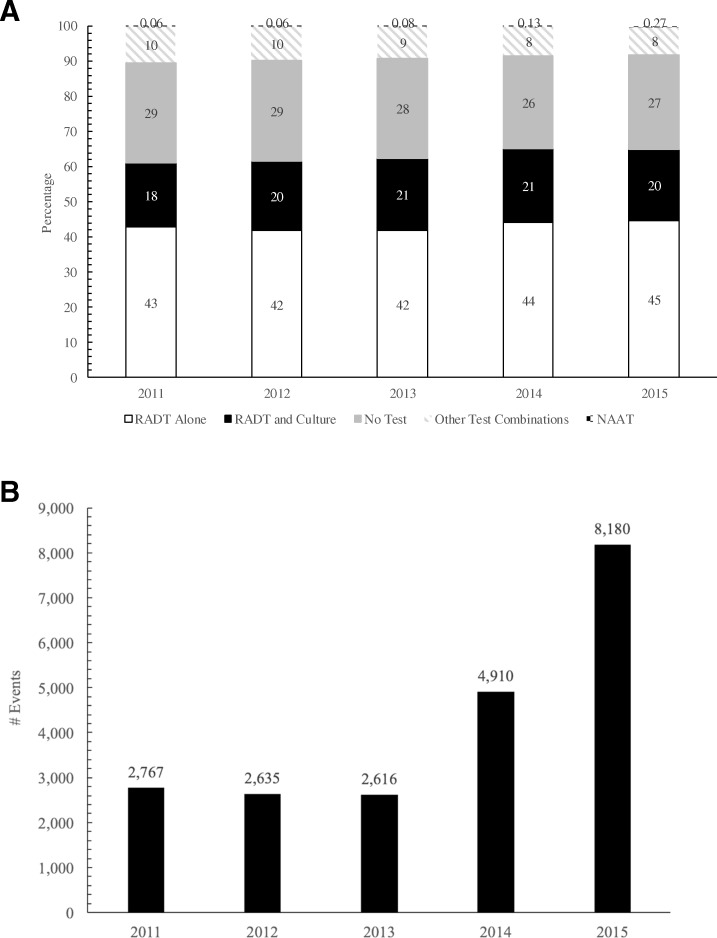
GAS Testing Patterns by Year: (**a**) proportions of all test types and (**b**) number of NAAT tests. NAAT, nucleic acid amplification testing; RADT, rapid antigen detection test

The most common place of service for pharyngitis events was the physician office (83.7%), with urgent care (7.0%) and emergency department (3.6%) being less common. Among all pharyngitis events, diagnosis by RADT was most common (43.0%), followed by no test (27.9%) and RADT plus culture (19.8%). Over the study period, the proportions of events diagnosed using RADT, RADT plus culture, no test, or other tests remained stable (Fig. 2a, *p* < 0.001). In contrast, the proportion of events diagnosed using NAAT, while small in overall percentage and number of cases, increased approximately 3.5-fold, from 0.06% in 2011 (*n* = 2767) to 0.27% in 2015 (*n* = 8180) (Fig. 2b, b, *p* < 0.0001).

RADT alone was the most common test type used by pediatricians (46.8%) and family physicians (45.8%). Pediatricians also had the highest proportion of events (among all events) diagnosed using RADT plus culture (32.6%; Additional file 1: Figure S1A, *p* < 0.001). In contrast, internists had the highest proportion of events diagnosed where no test was performed (45.6%). Similar testing patterns were observed when testing was stratified by place of service (Additional file 1: Figure S1B, *p* < 0.001), with office-based events showing the highest proportion of RADT only (45.7%), followed by no test (26.0%) and RADT plus culture (20.3%). Events involving the emergency department had no test in the majority of cases (53.7%). The proportion using NAAT was low (< 0.3%) among all provider specialties and places of service.

In a multivariable analysis, 28 and 40% of adult and pediatric NAAT claims, respectively, were associated with a laboratory/other setting (*p* < 0.0001). NAAT was significantly less likely to be used in the emergency department and urgent care (*p* < 0.0001 for both) than in an office setting (Additional file 2: Table S1).

To examine the differences in antibiotic prescription by age group, separate multivariable models were created for adult and pediatric patients. In both models, events diagnosed using RADT alone or no test were significantly associated with increased antibiotic prescription compared with events diagnosed using NAAT or RADT plus culture (Table 3). The majority of the cases diagnosed using RADT plus culture with antibiotic prescriptions had the treatment initiated on the day of the visit (66%). The use of RADT alone resulted in more antibiotic prescriptions compared with NAAT alone by 49% among patients aged ≥18 years (*p* < 0.0001) and 123% among patients aged < 18 years (*p* < 0.0001).

**Table 3:** Factors associated with antibiotic use among sore throat/pharyngitis visits^c^

	Events (n)	ABX %	Adjusted HR^b^	95% Confidence Limits	
A. 17 years and younger					
Diagnostic test (ref. NAAT)					
RADT only	4,682,423	52.49	2.23	2.16	2.31
RADT and culture	2,751,575	26.56	0.91	0.88	0.94
No test	1,834,351	55.73	2.30	2.22	2.38
Other test combinations	950,367	40.68	1.57	1.52	1.62
Place of service (ref. office)					
ED	262,364	50.15	1.01	1.00	1.01
Urgent care^a^	483,304	55.71	1.15	1.15	1.16
Laboratory/other	554,423	34.45	0.76	0.76	0.77
Provider type (ref. pediatrician)					
Family medicine	1,577,559	56.53	1.40	1.40	1.41
Internal medicine	235,431	55.6	1.40	1.39	1.41
Other	3,309,058	46.52	1.20	1.20	1.21
B. 18 years and older					
Diagnostic test (ref. NAAT)					
RADT only	3,400,327	54.66	1.49	1.44	1.54
RADT and culture	967,958	44.58	1.16	1.13	1.20
No test	3,397,174	57.88	1.59	1.54	1.64
Other test combinations	773,114	50.57	1.41	1.36	1.45
Place of service (ref. office)					
ED	411,369	51.01	0.92	0.91	0.92
Urgent care	824,376	57.39	1.10	1.10	1.11
Laboratory/other	537,543	41.82	0.73	0.72	0.73
Provider type (ref. family medicine)					
Internal medicine	235,431	55.6	0.99	0.99	0.99
Pediatrician	5,107,500	39.92	0.65	0.65	0.66
Other	3,309,058	46.52	0.93	0.92	0.93

Multivariable Anderson-Gill Survival Models and Adjusted HRs for Antibiotic Use in Patients Aged (A) < 18 Years and (B) ≥ 18 Years

^a^Urgent care center’s generally see patients on a first-come, first-serve basis for issues that require immediate attention but are not serious enough to warrant an emergency department visit

^b^Anderson and Gill’s Cox Regression Model Counting process using PROC PHREG (Reference)

^c^Adjusted for all variables in the table as well as patient’s sex, region, health plan, and calendar year. *P*-values< 0.0001 for all covariates except for *p* = 0.0031 for ED in patients < 18

*CI* confidence interval, *ED* emergency department, *HR* hazard ratio, *NAAT* nucleic acid amplification testing, *RADT* rapid antigen detection test

## Discussion

The current study evaluated the diagnosis and management of acute pharyngitis in the United States from 2011 through 2015 using a database of private and public health insurance claims. With over 11 million adult and pediatric patients with pharyngitis evaluated over the previous 5 years, these data represent one of the largest evaluations of GAS pharyngitis incidence, diagnosis, and antibiotic use to date and offer insights into real-world treatment practices among different types of providers across the United States. As would be expected, the majority of patients were cared for in office settings; family physicians or internists managed the majority of patients aged ≥18 years, and pediatricians managed the majority of patients aged < 18 years.

The diagnosis of pharyngitis was performed using a wide range of tests among different practitioner types and treatment settings. Overall, RADT alone was used in most cases (43%), likely given its ease of use and convenience. Additionally, no testing was done more frequently than RADT with confirmatory culture (27.9% vs 19.8%, respectively). These findings are in contrast to current clinical guidelines and may reflect real-world clinical practice. For example, clinics and emergency departments may face time pressures, patient demands, and delayed culture results, which may lead them to use RADT only. Alternatively, many cases of pharyngitis may appear obvious to the provider, resulting in no diagnostic testing at all. These real-world situations may contribute to incorrect GAS pharyngitis diagnosis, and consequently inappropriate antibiotic use, given the overlap of symptoms between viral and bacterial causes of pharyngitis. Of interest, while the overall use of NAAT alone was infrequent, the proportion of events diagnosed using NAAT vs. other test types increased 3.5-fold over the study period and roughly doubled in 2014 and again in 2015, coinciding with the approval of GAS pharyngitis NAAT assays.

Similar to results of other studies, almost 50% of patients in the present study received antibiotics for sore throat/pharyngitis [6, 7, 20]. Given that the literature indicates a 5–30% prevalence of GAS for pharyngitis [2, 4], this confirms high rates of unnecessary antibiotic use. Of interest, cases diagnosed by RADT alone or no test showed significantly greater antibiotic use than those diagnosed by NAAT alone. In contrast, NAAT alone had roughly equivalent rates of antibiotic prescription to those with the current gold standard of RADT plus culture. These results suggest that optimal testing practices that maximize diagnostic sensitivity may lead to more judicious antibiotic prescribing. They also may indicate that providers following current guidelines for diagnosis are more likely to prescribe in line with guidelines recommendations, limiting the empiric use of antibiotics.

FDA-cleared Strep A NAAT assays are now available, including Clinical Laboratory Improvement Amendments–waived PCR systems for use at the point of care (POC) that produce results within 15–25 min, with equivalent sensitivity and specificity to those of reference culture and/or laboratory-based PCR [15, 21]. As such, the use of these assays is possible at POC locations, such as physician offices, emergency departments, and urgent care clinics. Recent research has demonstrated that many patients prefer to avoid unnecessary antibiotics and are willing to undergo diagnostic tests at the POC to guide proper treatment [22]. Our results suggest that these POC NAAT assays could help minimize unnecessary antibiotic prescribing for acute pharyngitis/sore throat by removing the need for confirmatory culture testing, thus reducing the time for (and costs of) result reporting and additional healthcare contacts/calls between the patient and provider. Furthermore, based on the guidelines patients receiving RADT plus culture are likely RADT negative yet 66% of the antibiotic prescriptions for this group were still initiated at the time of the visit before culture results were available. This further emphasizes the potential value of highly sensitive and specific POC NAAT assays to aid clinician diagnosis and reduce empiric antibiotic prescriptions. Diagnostic systems utilizing NAAT technology have demonstrated sensitivity and specificity in clinical trials, but it is important to note that each diagnostic system is unique. Validation of all NAAT is important to ensure performance.

The careful selection of patients based on appropriate clinical symptoms for GAS testing is important regardless of testing modality [23]. A limitation of the analysis is lack of data on the Centor score, so it is unknown what proportion were eligible for GAS testing based on clinical symptoms. Diagnostic tests are always an aid to support clinician decision making. Due to increased sensitivity compared to RADT and even culture, a concern about the use of NAAT is increased detection of colonized patients who do not require treatment, which may theoretically encourage inappropriate antibiotic use of colonized patients. This reinforces the importance of physician assessment to select patients appropriate for GAS testing based on clinical criteria.

Although a strength of the current analysis is the large patient sample and database size, the results are limited by the types of data available and resultant limitations of the study methods and analyses. We acknowledge that the study population was pulled from a large convenience sample that disproportionately represents the Southern portion of the United States and those with insurance coverage by large employers. As such, our results may reflect regional and/or insurer-type restrictions that may be generalizable only to an employed and insured population. In addition, the data reflect only whether or not the patient received a service (e.g., visit for pharyngitis: yes or no; antibiotic prescribed: yes or no) and do not contain test results or clinical outcomes, which limits the study to one of association and not of causal pathways. As events with only rapid tests could have been from correctly identified positive cases, it is not known what proportion of events with both a rapid test and culture were from negative cases on rapid tests or missed positive cases on rapid tests. Similarly, events with no test may have been clear-cut clinical cases that were either very likely to be GAS pharyngitis or not GAS pharyngitis at all.

## Conclusions

Over a 5-year period in the United States, patient visits for acute pharyngitis events were common, often diagnosed by RADT alone or with no diagnostic test, and resulted in antibiotic prescription in roughly 50% of cases. Compared with patients < 18 years of age, patients ≥18 years of age were more likely to not receive any diagnostic test and be treated with antibiotics. In addition, NAAT use, while relatively uncommon, increased in frequency over the study period and resulted in antibiotic prescription rates similar to that with RADT plus confirmatory culture testing. Increasing awareness about the impact of antibiotic resistance requires revisiting the role of empirical antibiotic therapy. Diagnostics, in particular sensitive and specific POC diagnostics, can help clinicians to avoid prescribing unnecessary antibiotics. As such, the use of POC NAAT assays or RADT plus confirmatory culture testing may help to limit the inappropriate use of antibiotics and development of bacterial resistance.

These real-world data suggest that clinical guidelines should consider the role of newer diagnostic methods such as NAAT to improve the accuracy of GAS pharyngitis diagnosis as well as stress the overall value of diagnostic use along with clinical symptoms to confirm bacterial infections that require antibiotic treatment.

## Additional files

Additional file 1: **Figure S1.** GAS Testing by Provider Type: (A) provider specialty and (B) place of service. Other provider types include nurse practitioner/physician assistant, emergency medicine, otolaryngology, multiple providers, and unknown. ED, emergency department; NAAT, nucleic acid amplification testing; RADT, rapid antigen detection test. (DOCX 4476 kb)

Additional file 2: **Table S1.** Factors Associated With NAAT Use in Diagnosis of GAS Pharyngitis. Multivariable Anderson-Gill Survival Models and Adjusted HRs for NAAT Use in patients Aged (A) < 18 Years and (B) ≥ 18 Years. (DOCX 17 kb)

## Abbreviations

CCAE: Commercial claims and encounters
CDHP: Consumer-driven health plan
CI: Confidence interval
CLIA: Clinical laboratory improvement amendments
CPT: Current procedural terminology codes
Cx: Culture
ED: Emergency department
EPO: Exclusive provider organization
FDA: Food and drug administration
GAS: Group A streptococcus
HDHP: High-deductible health plan
HMO: Health maintenance organization
HR: Hazard ratios
MDCR: Medicare supplemental databases
NAAT: Nucleic Acid amplification testing
PCR: Polymerase chain reaction
POC: Point of care
POS: Point of service
PPO: Preferred provider organization
RADT: Rapid antigen detection test
SD: Standard deviation
US: United States
