# Supplementary figures and images for: Diagnosis and Management of Group a Streptococcal Pharyngitis in the United States, 2011–2015
Source: BMC Infect Dis. 2019 Feb 26;19:193. doi: 10.1186/s12879-019-3835-4 (PMC6390592; doi:10.1186/s12879-019-3835-4)

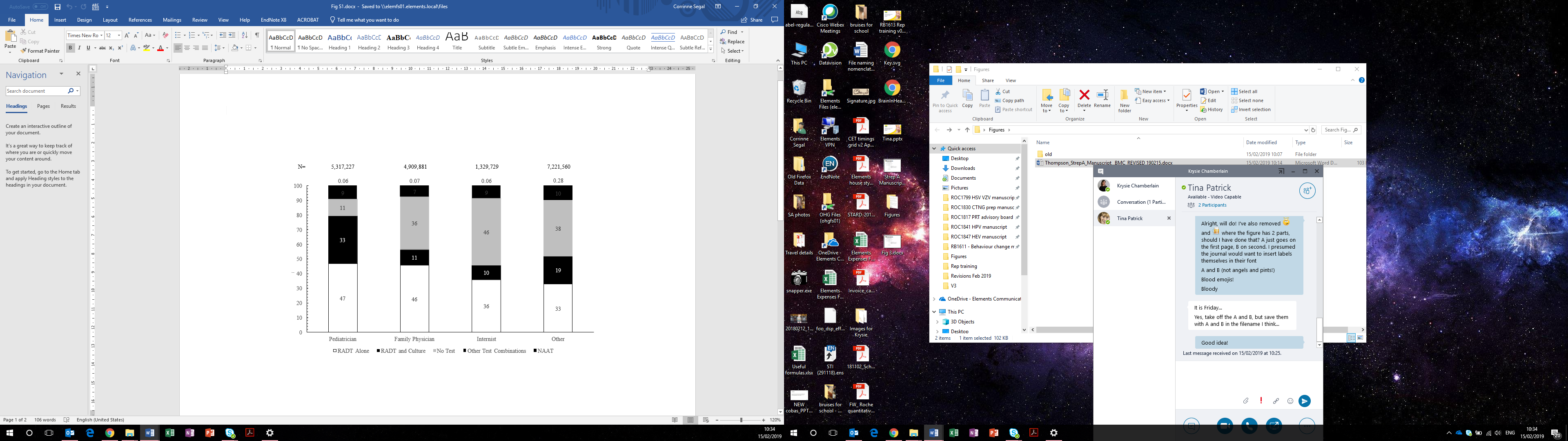


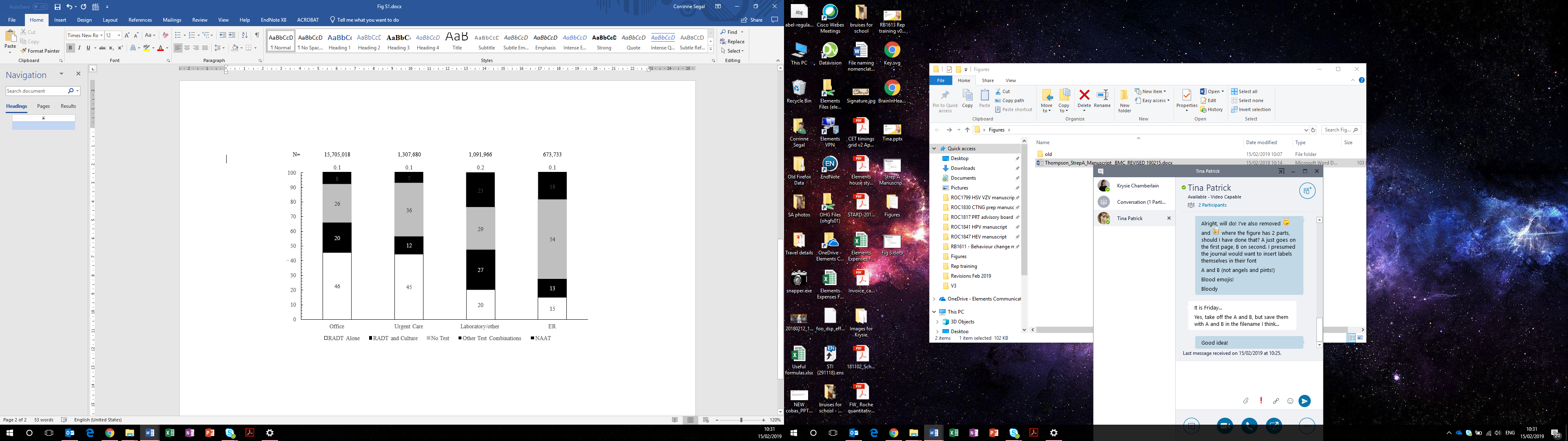

Supplement: Supplementary file 1 — Figure S1. GAS Testing by Provider Type: (A) provider specialty and (B) place of service. Other provider types include nurse practitioner/physician assistant, emergency medicine, otolaryngology, multiple providers, and unknown. ED, emergency department; NAAT, nucleic acid amplification testing; RADT, rapid antigen detection test. (DOCX 4476 kb) [file 12879_2019_3835_MOESM1_ESM.docx]
