# Supplementary material for: Diagnosis and Management of Group a Streptococcal Pharyngitis in the United States, 2011–2015
Source: BMC Infect Dis. 2019 Feb 26;19:193. doi: 10.1186/s12879-019-3835-4 (PMC6390592; doi:10.1186/s12879-019-3835-4)
Supplement: Supplementary file 2 — Table S1. Factors Associated With NAAT Use in Diagnosis of GAS Pharyngitis. Multivariable Anderson-Gill Survival Models and Adjusted HRs for NAAT Use in patients Aged (A) < 18 Years and (B) ≥ 18 Years. (DOCX 17 kb) [file 12879_2019_3835_MOESM2_ESM.docx]

|  |  | |  | | | |  | |  | |  |
| --- | --- | --- | --- | --- | --- | --- | --- | --- | --- | --- | --- |
|  | NAAT (n) | | Adjusted HR** | | 95% Confidence Limits | p-value |  |  |  | |  |
| A. 17 years and younger |  |  | |  |  |  |  |  | |  | |
| Place of service (ref. office) |  |  | |  |  |  |  |  | |  | |
| ED | 394 | 0.76 | | 0.68 | 0.84 | <.0001 |  |  | |  | |
| Urgent care | 822 | 0.75 | | 0.70 | 0.81 | <.0001 |  |  | |  | |
| Laboratory/other | 1,331 | 1.40 | | 1.31 | 1.48 | <.0001 |  |  | |  | |
| Provider type (ref. pediatrician) |  |  | |  |  |  |  |  | |  | |
| Family medicine | 1,104 | 1.08 | | 1.00 | 1.16 | 0.0393 |  |  | |  | |
| Internal medicine | 141 | 1.05 | | 0.88 | 1.25 | 0.5734 |  |  | |  | |
| Other | 6,949 | 3.22 | | 3.07 | 3.38 | <.0001 |  |  | |  | |
|  |  |  | |  |  |  |  |  | |  | |
| B. 18 years and older |  |  | |  |  |  |  |  | |  | |
| Place of service (ref. office) |  |  | |  |  |  |  |  | |  | |
| ED | 392 | 0.43 | | 0.38 | 0.48 | <.0001 |  |  | |  | |
| Urgent care | 824 | 0.47 | | 0.43 | 0.50 | <.0001 |  |  | |  | |
| Laboratory/other | 1,290 | 1.28 | | 1.20 | 1.36 | <.0001 |  |  | |  | |
| Provider type (ref. family medicine) |  |  | |  |  |  |  |  | |  | |
| Internal medicine | 766 | 0.97 | | 0.89 | 1.06 | 0.4973 |  |  | |  | |
| Pediatrician | 125 | 1.32 | | 1.13 | 1.55 | 0.0005 |  |  | |  | |
| Other | 7,433 | 2.77 | | 2.64 | 2.91 | <.0001 |  |  | |  | |
|  |  |  | |  |  |  |  |  | |  | |
| * Anderson and Gill's Cox Regression Model Counting process using PROC PHREG (Reference) | | | | |  |  |  |  | |  | |
| ** Adjusted for all variables in the table as well as patient's sex, region, health plan, calendar year | | | | |  |  |  |  | |  | |
| CI, confidence interval; Cx, bacterial culture and sensitivity testing; ED, emergency department; HR, hazard ratio; NAAT, nucleic acid amplification testing. | | | | | | | | | | | |
